# P-1197. NDM-5 and Siderophore Receptor Mutations Drive High Level Cefiderocol Resistance in Klebsiella pneumoniae: A Case Series

**DOI:** 10.1093/ofid/ofaf695.1390

**Published:** 2026-01-11

**Authors:** Michelle Potter, Wajih Askar, Gerardo F Gomez-Abundis, Kent Carpenter, Emir Kobic

**Affiliations:** Banner University Medical Center Phoenix, Phoenix, Arizona; Banner University Medical Center Phoenix, Phoenix, Arizona; Banner University Medical Center Phoenix, Phoenix, Arizona; Banner University Medical Center Phoenix, Phoenix, Arizona; Banner University Medical Center Phoenix, Phoenix, Arizona

## Abstract

**Background:**

Infections with Carbapenem-resistant Enterobacterales (CREs) are a serious public health threat. The emergence of distinct New Delhi metallo-beta-lactamase (NDM)-producing *K. pneumoniae* strains resistant to cefiderocol is a significant concern given the limited armamentarium for these carbapenemases.

**Table 1:**
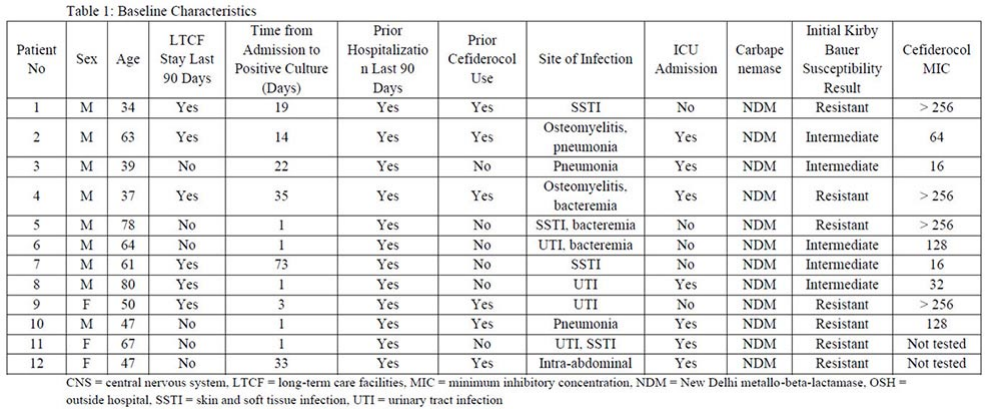
Baseline Characteristics CNS = central nervous system, LTCF = long-term care facilities, MIC = minimum inhibitory concentration, NDM = New Delhi metallo-beta-lactamase, OSH = outside hospital, SSTI = skin and soft tissue infection, UTI = urinary tract infection

**Table 2:**
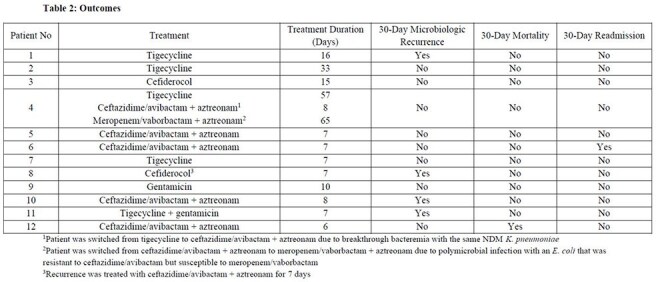
Outcomes 1Patient was switched from tigecycline to ceftazidime/avibactam + aztreonam due to breakthrough bacteremia with the same NDM K. pneumoniae 2Patient was switched from ceftazidime/avibactam + aztreonam to meropenem/vaborbactam + aztreonam due to polymicrobial infection with an E. coli that was resistant to ceftazidime/avibactam but susceptible to meropenem/vaborbactam 3Recurrence was treated with ceftazidime/avibactam + aztreonam for 7 days

**Methods:**

A case series of 12 patients was described from a single institution that had cefiderocol-resistant, NDM-producing *K. pneumoniae* infections between January 2023 and July 2024. Whole genome sequencing was performed to analyze resistance mechanisms, as well as mutations in siderophore transporters and porins.

**Figure:**
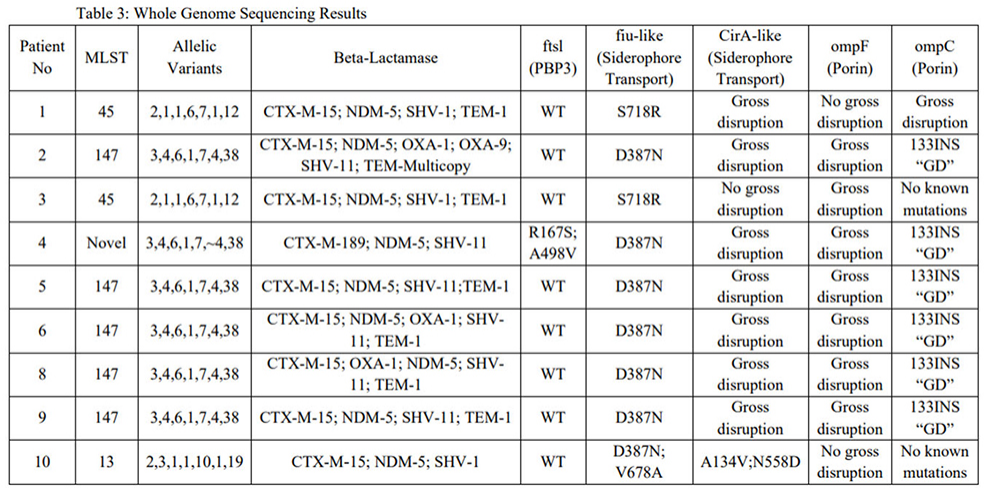
Whole Genome Sequencing Results

**Results:**

Patients presented with various infections, including skin and soft tissue infections, pneumonia, and bacteremia. Of concern, cefiderocol resistance was seen among patients with and without prior cefiderocol exposure. Genomic analysis for 9 patients revealed NDM-5 in every isolate, along with additional mutations associated with resistance. Treatment with ceftazidime-avibactam plus aztreonam was successful in most instances, although microbiologic recurrence occurred in certain cases.

**Conclusion:**

High level cefiderocol-resistance among NDM-5 producing *K. pneumoniae* with siderophore mutations add more challenges to treating CRE infections. Stricter infection control measures along with enhanced surveillance are needed, especially in regions where NDM is endemic to limit additional spread of these variants.

**Disclosures:**

Emir Kobic, BCIDP, Shionogi: Honoraria

